# Constipation and the Quality of Life in Conservatively Treated Chronic Kidney Disease Patients: A Cross-sectional Study

**DOI:** 10.7150/ijms.49648

**Published:** 2020-10-18

**Authors:** Jakub Ruszkowski, Zbigniew Heleniak, Ewa Król, Agnieszka Tarasewicz, Joanna Gałgowska, Jacek M. Witkowski, Alicja Dębska-Ślizień

**Affiliations:** 1 Department of Pathophysiology, Faculty of Medicine, Medical University of Gdańsk, Gdańsk, Poland; 2 Department of Nephrology, Transplantology and Internal Medicine, Faculty of Medicine, Medical University of Gdańsk, Gdańsk, Poland

**Keywords:** chronic kidney disease, constipation, flatulence, SF-36v2

## Abstract

**Background:** Constipation is a common gastrointestinal disorder that in general population is associated with worse health-related quality of life (HRQoL). The epidemiology of constipation has not been reliably determined in conservatively-treated CKD patients. We aimed to determine the prevalence of constipation and constipation-related symptoms in conservatively-treated CKD patients, to find factors associated with their altered prevalence ratio (PR), and to verify the associations between constipation and HRQoL.

**Methods:** In this cross-sectional study, 111 conservatively-treated CKD outpatients fulfilled questionnaires that included questions addressing HRQoL (SF-36v2®), constipation-related symptoms (The Patient Assessment of Constipation‐Symptoms questionnaire), the Bristol stool form scale (BSFS), Rome III criteria of functional constipation (FC), and frequency of bowel movement (BM).

**Results:** Depending on the used definition, the prevalence of constipation was 6.6-28.9%. Diuretics and paracetamol were independently associated with increased PR of BSFS-diagnosed constipation (PR 2.86, 95% CI 1.28-6.37, *P* = 0.01) and FC (PR 2.67, 95% CI 1.07-6.64, *P* = 0.035), respectively. The most commonly reported symptoms were bloating (50.9%) and straining to pass a BM (42.7%). Abdominal discomfort (37.3%) was independently associated with worse scores in all analyzed HRQoL domains. In multiple regressions, FC and having <7 BM/week, but not BSFS-diagnosed constipation, were associated with lower scores in several HRQoL domains.

**Conclusions:** Constipation and related symptoms are prevalent in CKD patients. FC and decreased frequency of defecation, but not BSFS-diagnosed constipation, are associated with worse assessment of HRQoL in conservatively-treated CKD patients.

## Introduction

Constipation is a common gastrointestinal problem. In the general population of Europe, the mean value of the reported constipation prevalence is 17.1%, and its higher value is frequently associated with older age, female sex, less self-reported physical activity, and certain medications 1, 2. In general population, constipation is associated not only with worse health-related quality of life (HRQoL) 3, 4 but also with higher risk of cardiovascular events and all-cause mortality 5-7.

Chronic kidney disease (CKD), that is defined as abnormalities of kidney structure or function, present for >3 months, with implications for health 8, is a condition affecting approximately 9.1-13.4% of the global population 9, 10. The severity of CKD is divided into 5 stages (G1-G5) according to the level of glomerular filtration rate (GFR). Recent studies suggest a two-way relationship between constipation and CKD. On the one hand, the presence of constipation is independently associated with a higher risk of developing CKD and end-stage renal disease (ESRD); it is hypothesized that these relationships can be mediated by an altered gut microbiota and/or be a result of common cause such as lack of regular physical activity 11-13. On the other hand, CKD since its moderate stage (i.e. G3b, eGFR < 45 ml/min/1.73 m^2^) is known to be associated with several upper gastrointestinal symptoms such as loss of appetite and vomiting 14. As the disease progresses, the prevalence of the gastrointestinal (GI) symptoms increases 14, 15. Among all GI symptoms, constipation is the most frequently assessed GI symptom in dialysis patients, and the prevalence of constipation is higher in hemodialysis (HD) patient than in peritoneal dialysis (PD) patients (23.8-71.7% vs 14.2-28.9% of patients, respectively) 16. In dialysis patients, like in a general population, constipation is associated with worse HRQoL 17.

However, there is little known on the epidemiology of constipation and related symptoms in patients in the earlier stages of CKD (non-ESRD) 18. The present study aimed to determine the prevalence of constipation and constipation-related symptoms in conservatively-treated CKD patients, to find factors associated with their altered prevalence ratio (PR), and to verify the associations between the occurrence of constipation and HRQoL.

## Materials and Methods

This cross-sectional study was carried out within the time frame of June 2018 and December 2019. We recruited a total of 111 outpatients that were visiting Nephrological Outpatient Clinic, a part of the University Clinical Centre in Gdańsk. Ethical permission for the study was obtained from the Bioethical Committee for Scientific Research at the Medical University of Gdańsk (NKBBN/426-56/2018).

The participants were selected according to the following criteria: diagnosis of CKD, age above 18 years, voluntary participation. Exclusion criteria were receiving currently or in the past dialysis, kidney transplantation, cognitive deficits and visual impairment that unable of answering the questionnaire; having a serious illness in an acute treatment phase. All patients were informed about the nature and purposes of the study. As the research was based on the voluntarily filled anonymous surveys, additional written informed consents were unnecessary and were not collected.

All included participants were asked to voluntarily complete questionnaires that included a battery of surveys: (1) addressing the HRQoL: the Polish versions of the SF-36v2® Health Survey (SF-36v2); (2) addressing symptoms of constipation: a question about the number of defecation per week, The Patient Assessment of Constipation‐Symptoms (PAC‐SYM) questionnaire 19, simple questions containing Rome III criteria of functional constipation (FC) 20, and a request to select the most common stool consistency on the Bristol stool form scale (BSFS) 21, 22.

The physician completed a questionnaire that included multiple-choice questions about comorbidities (diabetes, hypertension, heart failure, hypothyroidism, depression, inflammatory bowel disease) and taken medications (iron, calcium, vitamin D, antihistamines, calcium channel blockers, beta-blockers, diuretics, hypnotics, tricyclic antidepressants, nonsteroidal anti-inflammatory drugs, paracetamol, lactulose, other laxatives, probiotics, oral contraceptives). Data on sex, age, body weight, height, estimated glomerular filtration rate (eGFR) based on CKD-EPI formula, and etiology of CKD were collected. The body mass index (BMI) was calculated as the body weight divided by the square of the body height.

To use the SF-36v2, a non-commercial license agreement was made between JR and OptumInsight Life Sciences, Inc. (license number: QM044526). Validation and scoring of SF-36v2 were performed using desktop scoring software PRO CoRE Version 1.4 provided by Optum. The SF-36v2 questionnaire consists of 36 items that measure eight dimensions of HRQoL: physical functioning (PF); role limitations due to physical health problems (RP); bodily pain (BP); general health perception (GH); vitality (VT); social functioning (SF); role limitations due to emotional problems (RE); and mental health (MH). All dimensions are scored such that higher scores indicate better HRQoL. Firstly, the reliability and validity of subscales were tested (Supplementary material, *Table S1*). Only the GH subscale was neither reliable (Cronbach's alpha = 0.65) nor valid. This finding is supported by Żołnierczyk-Zreda who found that all subscales of the Polish version of SF-36v2, besides GH (Cronbach's alpha = 0.63), are reliable in general population 23. Based on the poor reliability and validity of GH, it was excluded from further analysis.

To use the PAC-SYM, a non-commercial license agreement was made between JR and Mapi Research Trust (license number: 10328). The questionnaire contains 12 items assessing the severity of abdominal, rectal, and stool symptoms of constipation 19. Items are scored on 5‐point Likert scale (0 = “symptom absent”, 1 = “mild”, 2 = “moderate”, 3 = “severe”, and 4 = “very severe”). In statistical analysis, due to small number of patients reporting “severe” or “very severe” symptoms, they were counted together with patients reporting “moderate” severity of symptoms.

In the context of BSFS, constipation was defined as either type 1 (“Separate hard lumps, like nuts (difficult to pass)”) or type 2 (“Sausage-shaped but lumpy”) stool form. For the diagnosis of FC, the presence of at least 2 out of 6 symptoms (straining; lumpy or hard stools; sensation of incomplete evacuation; sensation of anorectal obstruction or blockage during defecation; less than three bowel movements per week; need for manual maneuvers to facilitate defecation) in at least 25% of the defecations, for at least 3 months, with symptom onset at least 6 months before diagnosis, had been met 20.

### Statistical analysis

Testing the normality of the distribution of collected data was performed using the Shapiro-Wilk test. Continuous variables with non-normal distribution were presented using medians and interquartile ranges (IQR). Categorical variables were presented as a percentage share of the obtained data. Patient groups were compared using Mann-Whitney *U*, Kruskal-Wallis ANOVA, and Pearson's chi-squared (χ^2^) tests. Statistical testing was done with Statistica, v.13.0 (StatSoft Polska, Inc. 2016) and Python libraries: Pandas 24, Pingouin 25, Statsmodels 26. *P* values < 0.05 were considered significant. *P* values for multiple comparisons were adjusted using Hommel method. Figure 2 was designed in Microsoft Excel 2013.

Due to high disproportion in number of patients across stages of CKD, patients were divided into 3 groups according to eGFR terciles: with low eGFR (≤ 32 ml/min/1.73 m²), medium eGFR (33-43 ml/min/1.73 m²), and high eGFR (≥44 ml/min/1.73 m²). Correlations between eGFR terciles and symptoms severities were presented as Goodman-Kruskal gamma coefficient, and their significance was tested with *Z*-test.

As several variables were suspected to be associated with constipation, we used Poisson regression models with robust variance (computed with Statsmodels adaptation of R code published by Gallis and Turner 27) to estimate prevalence ratio (PR) simultaneously controlling for multiple variables. Potential predictors of constipation occurrence were age, BMI, sex, eGFR terciles, comorbidities (all besides hypertension as it is not associated with altered gastrointestinal motility in the literature), and taking specific medications. All variables were initially tested in univariate Poisson regression models with robust variance. If *P* value of model testing the association between taking a drug and constipation was lower than 0.20, this variable was selected for inclusion into a single Poisson regression model with all variables related to demographics and comorbidities. If the interaction between sex and age was statistically significant, it was added to the multiple regression model.

To determine whether constipation or constipation-related symptoms were independently associated with altered score in any of the HRQoL domain, multivariable ordinary least squares regressions were applied. Each of the models was adjusted to sex, age, BMI, eGFR tercile and comorbidities. Such models were shown if both GI symptom coefficient significantly differs from zero (*T*-test) and its adding to model significantly improves model (ANOVA *F*-test). To select final model for each HRQoL domain, among models with all combinations of GI symptoms from the previous step, one was chosen based on Akaike's information criterion that aims to balance goodness‑of‑fit and model complexity.

## Results

We have screened 150 patients. The main exclusion criteria were as follows: visual impairment that made it impossible to complete the questionnaire; lack of consent without explaining the reason.

### Demographics and comorbidities

Demographics and comorbidities were presented in Table 1. Except for the higher frequency of hypothyroidism in women (26.5% vs men: 8.1%; unadjusted *P* = 0.009, adjusted *P* = 0.04), no other differences were found between sexes. Similarly, patients divided into groups based on terciles of eGFR did not differ between each other in manner of comorbidities nor BMI, but patients with medium eGFR were significantly older than those with high eGFR (adjusted *P* = 0.01; post-hoc *P* = 0.005).

### Constipation and related symptoms

**Figure 1:**
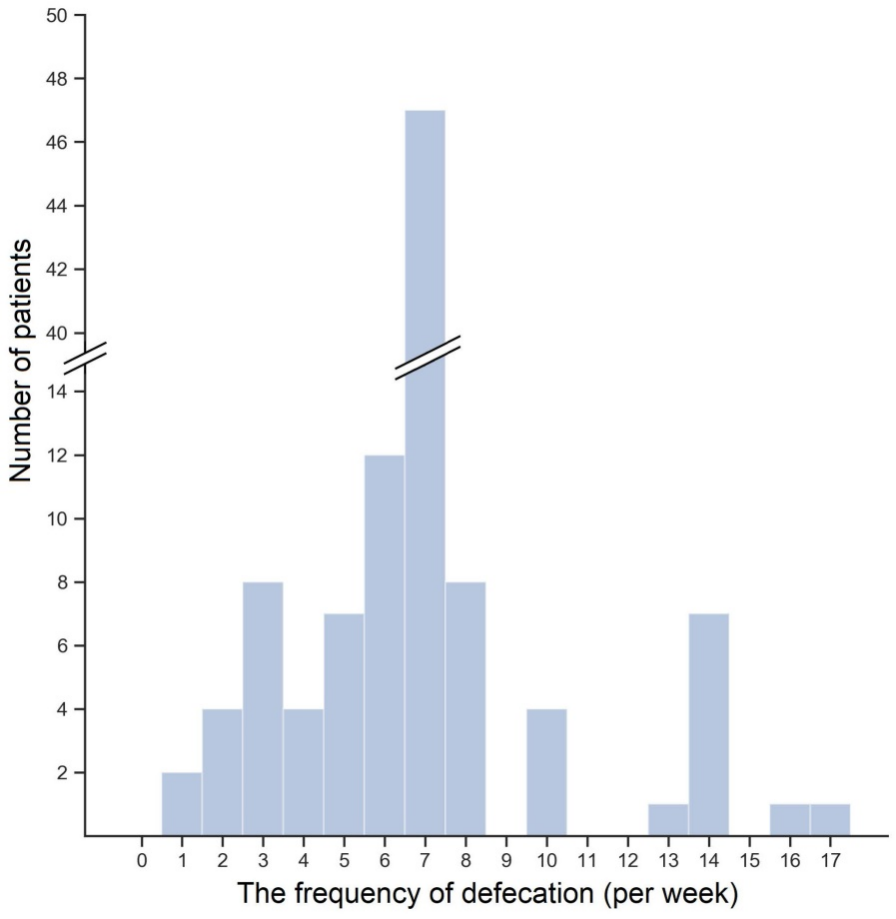
The histogram of the bowel movements frequency per week

The median number of defecation per week was 7 (IQR: 6-7). No differences were found in the frequency of defecation neither between genders nor among eGFR terciles (data not shown). In Figure 1, the distribution of bowel movements (BMs) frequency per week was shown. The majority of patients—43.4%—reported a mean seven BMs a week. Lower frequency of defecation occurred in 35.8% of patients, and 6.6% of patients reported frequency even lower than three BMs a week. In contrast, 20.8% of patients had BM more often than once a day. Interestingly, lower than mean 7 BMs per week was reported by 25.0%, 39.4%, and 43.2% of patients with high, medium, and low eGFR, but the observed difference was insignificant (*P* = 0.23).

**Figure 2:**
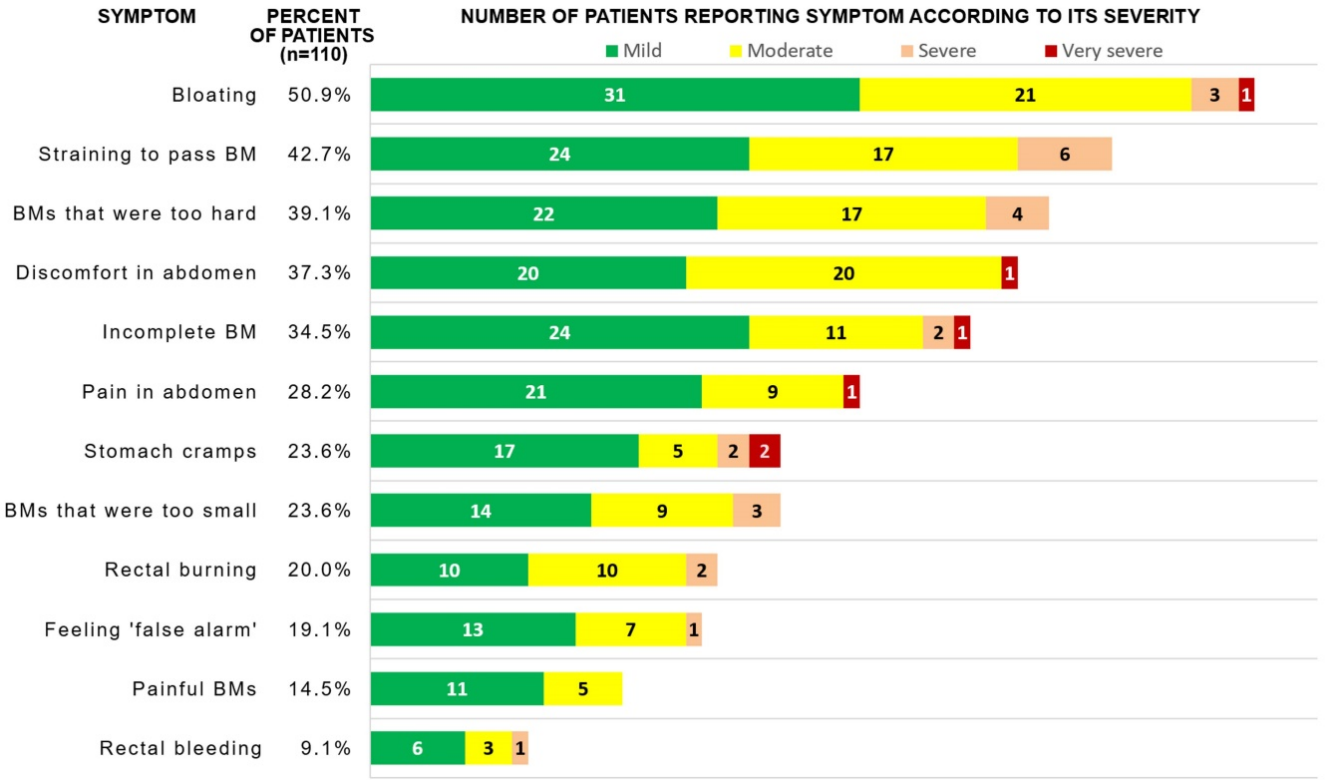
Frequency of symptom reporting and absolute number of patients reporting each severity of gastrointestinal symptoms in PAC-SYM scale

The most commonly reported symptoms in the PAC-SYM questionnaire were following: bloating (50.9%), straining/squeezing to pass BM (42.7%), too hard stool (39.1%), abdominal discomfort (37.3%), and feeling of incomplete BM (34.5%). If a symptom was reported, it was reported to be mild, moderate, severe and very severe in 56.5%, 35.5%, 6.4% and 1.6% of cases, respectively (see Fig. 2 for absolute numbers). After adjustment for multiple comparisons, patients with high, medium and low eGFR did not significantly differ in the frequency of constipation-related symptoms; however, terciles of eGFR did negatively correlate with severity of four symptoms (Table 2), i.e. patients with lower eGFR more frequently reported higher severity of the symptoms.

FC and constipation diagnosed with BSFS were found in 21 (18.9%) and 28 (28.9% of patients who completed the scale) of patients, respectively. Neither the above-mentioned symptoms nor any kind of constipation were associated with gender (data not shown).

Since constipation is a common side effect of certain drugs, we estimated prevalence ratio (PR) of constipation in patients according to their drug-use patterns, adjusted to demographics, eGFR tercile and comorbidities (see details in Methods). The frequency of drug-taking is summarized in Supplementary material, *Table S2*. As shown in Table 3, for constipation diagnosed with the BSFS, besides female sex and increasing age, taking diuretics was independently associated with increased PR of constipation (adjusted PR 2.86, 95% CI 1.28-6.37, *P* = 0.01). In contrast, paracetamol and low eGFR (≤ 32 ml/min/1.73 m^2^) were associated with increased PR of FC, whereas taking NSAIDs was independently associated with lower PR of FC (Supplementary material, *Table S3*). No drug was independently associated with having less than once BM a day (Supplementary material, *Table S4*).

### Health-related quality of life

SF-36v2 is a tool commonly used to assess the HRQoL that has been validated also in Poland 23. To evaluate associations between HRQoL and GI symptoms, we performed linear regressions that were adjusted for demographic and clinical data (columns 'Adjusted univariate analyses' in Table 4 and Supplementary material, *Tables S5-S10*). We found that the presence of discomfort in the abdomen was independently associated with worse scores in all HRQoL domains (Table 4 and Supplementary material, *Tables S5-S10*). Similarly, the presence of pain in the abdomen was associated with worse assessment of all, save RE, HRQoL domains (Table 4 and Supplementary material, *Tables S5-S9*). Also, painful BMs reporting was significantly associated with lower scores in MH, VT, SF, and RE (Table 4 and Supplementary material, *Tables S8-S10*). Defecation less frequently than mean once a day was associated with lower scores in PF, RP and MH (Table 4 and Supplementary material, *Tables S5-S6*). FC was associated with BP and VT (Supplementary material, *Tables S7-S8*). Interestingly, BSFS-diagnosed constipation was not associated with a lower score in any HRQoL domain.

To find out how many and which of the symptoms should be considered assessing the HRQoL, we have selected the balanced adjusted multiple regression model for each HRQoL domain (columns 'Selected adjusted multiple regression' in Table 4 and Supplementary material, *Tables S5-S10*). The following symptoms were in at least two models: pain in the abdomen (PF, RP, BP, SF), discomfort in the abdomen (VT, SF), altered frequency of defecation (PF, RP), having BMs that were too hard (BP, VT), too small (PF, RE) or painful (VT, RE). Such selected models explained 33.7-53.0% of the variability of the HRQoL scores (R^2^ reported in columns 'Selected adjusted multiple regression' in Table 4 and Supplementary material, *Tables S5-S10*), whereas models based only on sex, age, BMI, eGFR tercile and comorbidities explained only 13.6-34.6% of the variability.

## Discussion

Our study aimed to determine the prevalence of constipation-related symptoms in conservatively-treated CKD patients, as well as to verify the relationship between them and HRQoL. Using validated questionnaires, we found that a number of gastrointestinal symptoms are frequently reported by CKD patients, and that presence of symptoms was associated with worse HRQoL.

Since constipation prevalence varies widely due to differences in the used constipation definition 2, 28-30, we used three ways to define constipation: the BSFS, Rome III criteria, and the decreased frequency of BM per week. In the general population of Europe, the mean value of the reported constipation rates is 17.1% 2; that is a lower value than the prevalence of FC and BSFS-based constipation in our study; that is 18.9% and 28.9%, respectively. Recently published comprehensive review article about constipation in CKD revealed that despite strong evidence on higher prevalence of constipation in dialysis patients (23.8-71.7% and 14.2-28.9% of HD and PD patients, respectively 16), information is scarce on the epidemiology of constipation among patients with conservatively-treated patients 18*.* To our knowledge, up-to-date only two studies that included conservatively-treated CKD patients used Rome III criteria and BSFS to determine the prevalence of constipation 12, 30. In the first, FC and BSFS-diagnosed constipation were recognized in 5% and 19% of 21 non-dialysis ESRD patients, respectively. In the second, FC and BSFS-diagnosed constipation were recognized in 35% and 33% of 43 non-dialysis nondiabetic patients with eGFR <45 mL/min/1.73 m^2^, respectively. Since the number of included participants was limited, we agree with authors of the above-mentioned review article that there is a need for more data on constipation epidemiology among conservatively-treated CKD patients 18. We not only did determine the prevalence in more than twofold bigger population of patients, but also found factors associated with the altered prevalence of constipations.

Indeed, we found out that paracetamol and diuretics were independently associated with increased PR of constipation diagnosed with Rome III criteria and BSFS, respectively. Even though all these drugs have been associated with constipation in other patients populations 31-33, the mechanisms leading to such side-effects are not clear. Regarding paracetamol, Chang et al. suggested that its pro-constipation properties can be attributed to the anti-serotonergic effects of paracetamol; however, it was not investigated directly yet 32. It is hypothesized that diuretics can cause constipation secondary to dehydration, electrolyte disturbances, or, less probably, directly suppressing gut motility 33, 34. In the presented study, we confirmed associations between the presence of constipation and lower HRQoL. We have demonstrated that FC is independently associated with BP and VT, parts of SF-36v2 physical and mental summary components, respectively. Similarly, Zhang et al. have shown that FC is associated with lower scores in both physical and mental summary components of HRQoL in both HD and PD patients 17. Interestingly, our study has indicated that constipation diagnosed with BSFS is not associated with worse HRQoL in conservatively-treated CKD patients. It is highly interesting as previous studies did not perform such analyses. As the BSFS is a clinical surrogate of whole-gut and colonic transit 35, we hypothesize that the deterioration in HRQoL of constipated CKD patients is not a consequence of delayed gut transit. More probable, given the associations between functional constipation and decreased HRQoL, and following the knowledge about the pathophysiology of functional gastrointestinal disorders, decreased HRQoL might be a manifestation of a disturbance in bidirectional relationship of the gastrointestinal tract and nervous system 36.

Even though we have not seen an association between constipation diagnosed with BSFS and worse HRQoL, assessment of BSFS should not be neglected in CKD patients. Ramos et al. found that so defined constipation was associated with significantly higher serum concentration of *p*-cresyl sulfate, a microbial-derived uremic toxin 12. Interestingly, the same study failed to show an association between FC and the serum concentration of either *p*-cresyl sulfate or indoxyl sulfate. Since cardiovascular disease is the leading cause of death in CKD and there are data suggesting that constipation is associated with increased cardiovascular risk 5, 37, further studies are needed to compare clinical associations and cardiovascular mortality between CKD patients with constipation according to BSFS versus Rome criteria.

Using the PAC-SYM questionnaire, we have shown that the severity of straining to pass BM, as well as painful, incomplete, or too hard BM correlated with the deterioration of kidney function. That is, patients with low eGFR assessed the severity of these symptoms as at least moderate from 1.8 (straining to pass BM) to even 4 (incomplete BM) times more frequently than patients with high eGFR. Moreover, low eGFR was independently associated with a 2.85 times higher prevalence of FC in comparison to high eGFR. The high and low eGFR terciles in our study share high similarity to G1-G3a and G4-G5 CKD stages, respectively. Unfortunately, patients with G3b CKD were also present as a small fractions of upper tercile (8% of individuals in this tercile) and lower tercile (35% of individuals in this tercile), thus direct translation of the results into CKD stages is impossible due to high disproportion in the number of recruited patients across stages of CKD.

Knowing the prevalence of constipation and related symptoms and their deteriorating impact on HRQoL, it is important to find effective methods of lower gastrointestinal symptoms management for CKD patients. Firstly, nonpharmacological treatment should be considered, i.e. increase in physical activity and improvement of diet 38. Indeed, one can recommend a fiber-rich diet because it shortens intestinal transit time (via bulking effect 39) and, additionally, is a part of healthy dietary patterns that are associated with lower mortality in CKD patients 40. However, based on the high prevalence of bloating among CKD patients in our study, as well as that some CKD patients require fluid intake restriction, the recommendation of a diet rich in fiber should be given cautiously. In fact, the high-fiber diet can lead to exacerbation of flatulence (via retardation of intestinal gas propulsion 41)^42^, and water restriction reduces the pro-motile effect of fiber 43. Unfortunately, there are no clinical trials assessing the efficacy of diet modification on constipation reduction in conservatively-treated CKD patients. Interestingly, the consumption of 40 g of raw almonds daily for four weeks was safe and improved both BSFS-diagnosed constipation and HRQoL in HD patients 44. In view of our findings, similar trials are desirable in the population of conservatively-treated CKD patients.

The next step of constipation management is the introduction of pharmacotherapy 18. Since there is no data on the safety and efficacy of laxatives in CKD patients, one should take into account that some of them can have limited efficacy in patients restricting fluid intake (stool softeners, i.e. docusate sodium/calcium) and some may exacerbate constipation-related symptoms, e.g. flatulence (lactulose). As the majority of CKD patients in our study reported bloating, anti-foaming agents—simethicone and dimethicone—should be taken into account because they are effective in relieving abdominal distension and flatulence in functional gastrointestinal disorders 45, 46. Moreover, alleviation of abdominal bloating in constipated patients is achievable using new laxative drugs: lubiprostone (a type-2 chloride channel activator), linaclotide (a guanylate cyclase-C receptor agonist), prucalopride (selective 5-HT4 receptor agonist), and elobixibat (an ileal bile acid transporter inhibitor) 47-50.

Interestingly, according to animal studies, a part of drugs used in constipation treatment can possess nephroprotective properties. In adenine-induced CKD rat model, lactulose—a prebiotic disaccharide—was shown to possess nephroprotective properties (i.e. suppressed tubulointerstitial fibrosis) via reduction of microbiota-derived uremic toxin, indoxyl sulfate 51. Since lactulose reduces microbiota-derived uremic toxins, indoxyl sulfate and *p*-cresol, in humans 52, it can be hypothesized that introduction of such constipation treatment can additionally slow the progression of CKD. Similarly, lubiprostone and linaclotide can possess nephroprotective properties via improving the gut microbiota and intestinal environment as was demonstrated in adenine-induced CKD mouse model 53, 54. However, the nephroprotective properties of these laxatives should be confirmed in clinical trials with conservatively-treated CKD patients. Such studies could also determine whether the treatment of constipation significantly improves HRQoL in this population. In a multicenter, observational study of hemodialysis patients with FC, elobixibat significantly increased the frequency of BM and improved patients' HRQoL 55. Furthermore, based on mechanistic insights into the “gut-kidney-heart” axis, Sumida and Kovesdy have recently hypothesized that the administration of laxatives might be a gut microbiota-targeted therapeutic intervention for reduction cardiovascular risk in patients with CKD 37.

The relatively small number of surveyed patients, lack of healthy control group, lack of information about proteinuria, direct usage of Rome III criteria in authors' questions instead of validated diagnostic questionnaire to diagnose FC, and a cross-sectional character of the study can limit the importance of obtained results. However, our study possesses also considerable advantages such as comprehensiveness (detailing the prevalence of constipation and constipation-related symptoms, finding factors associated with the altered prevalence of constipation, and analysis of associations between the symptoms and HRQoL domains), inclusion of more than twofold bigger population than in previous similar studies, and use of the method of *P* values correction limiting the probability of false discovery (a type I error). Our study, as one of the first in the field, should prompt researchers to determine the epidemiology of constipation and related symptoms in conservatively-treated CKD patients, as well as to establish the biochemical, clinical and patient-oriented benefits of their treatment.

## Supplementary Material

Supplementary tables.

## Figures and Tables

**Table 1:** Demographic and clinical parameters of the total study population and according to eGFR tercile

	All	High eGFR tercile	Medium eGFR tercile	Low eGFR tercile	Unadjusted *P* value
**Participants, n**	111	37	34	40	-
**Male, n (%)**	62 (55.9)	21 (56.8)	12 (35.3)	29 (72.5)	0.006
**Age, years, median (IQR)**	68 (55.0-74.0)	64 (44.0-71.0)	71.0 (68.0-76.0)	66.0 (53.5-72.5)	0.005
**BMI, kg/m^2^, median (IQR)**	28.48 (25.63-31.14)	28.09 (25.18-30.76)	28.48 (26.23-30.49)	28.58 (24.49-31.92)	0.84
**eGFR, median (IQR)**	38.0 (30.0-48.0)	57.0 (48.0-67.0)	38.0 (35.0-42.0)	26.5 (17.0-31.0)	<0.001
**Hypertension, n (%)**	97 (87.4)	31 (83.8)	32 (94.1)	34 (85.0)	0.36
**Diabetes, n (%)**	35 (31.5)	6 (16.2)	13 (38.2)	16 (40.0)	0.05
**Heart failure, n (%)**	22 (19.8)	4 (10.8)	8 (23.5)	10 (25.0)	0.24
**Hypothyroidism, n (%)**	18 (16.2)	6 (16.2)	7 (20.6)	5 (12.5)	0.64
**Depression, n (%)**	5 (4.5)	0	3 (8.8)	2 (5.0)	0.20

Abbreviations: BMI: body mass index; CKD: chronic kidney disease; eGFR: estimated glomerular filtration rate

**Table 2:** The frequencies of constipation-related symptoms reporting by eGFR-grouped patients and the correlations between symptoms severities and eGFR terciles

Symptom and its severity		Percentage of patients reporting the symptom			Gamma coefficient	*P* value	
		High eGFR group (n = 36)	Medium eGFR group (n = 34)	Low eGFR group (n = 40)		Unadjusted	Adjusted
**Painful BM**	Lack	97.2%	85.3%	75.0%	-0.569	<0.001	0.002
	Mild	0.0%	11.8%	17.5%			
	At least moderate	2.8%	2.9%	7.5%			
**Straining to pass BM**	Lack	83.3%	82.3%	77.5%	-0.427	<0.001	<0.001
	Mild	11.1%	11.8%	12.5%			
	At least moderate	5.6%	5.9%	10.0%			
**Incomplete BM**	Lack	75.0%	73.5%	50.0%	-0.371	<0.001	0.006
	Mild	19.4%	17.7%	27.5%			
	At least moderate	5.6%	8.8%	22.5%			
**Too hard BM**	Lack	77.8%	52.9%	52.5%	-0.323	0.002	0.015
	Mild	11.1%	29.4%	20.0%			
	At least moderate	11.1%	17.6%	27.5%			

Abbreviations: BM: bowel movement; eGFR: estimated glomerular filtration rate

**Table 3:** Poisson regression models showing variables significantly and independently associated with prevalence ratio of constipation diagnosed with the Bristol Stool Form Scale

Variable	Univariate analyses (each row represents separate model)		Multiple regression	
	PR (95% CI)	*P* value	PR (95% CI)	*P* value
**Use of diuretics**	2.72 (1.21-6.14)	0.016	2.86 (1.28-6.37)	0.010
**Age, years**	1.015 (0.99-1.04)	0.231	1.053 (1.01-1.10)	0.012
**Female sex**	1.57 (0.83-2.97)	0.161	120.72 (2.54-5728.74)^a^	0.015
**Female sex × age interaction**	-	-	0.935 (0.89-0.99)	0.015
**BMI, kg/m^2^**	1.004 (0.94-1.07)	0.897	1.02 (0.96-1.09)	0.528
**eGFR tercile:**				
High	reference	-	reference	-
Medium	1.43 (0.54-3.75)	0.469	1.38 (0.58-3.33)	0.468
Low	1.30 (0.50-3.42)	0.592	1.74 (0.68-4.49)	0.250
**Diabetes**	1.05 (0.53-2.06)	0.891	0.81 (0.41-1.61)	0.546
**Heart failure**	1.09 (0.51-2.33)	0.832	0.84 (0.38-1.87)	0.667
**Hypothyroidism**	1.23 (0.55-2.73)	0.616	1.10 (0.50-2.37)	0.817
**Depression**	1.46 (0.47-4.48)	0.512	1.10 (0.31-3.89)	0.879

Abbreviations: BMI: body mass index; CI: confidence interval; eGFR: estimated glomerular filtration rate; PR: prevalence ratio ^a^ Due to significant interaction with age, being women at median age of 69 was associated with PR approx. 1.17 compared to men in this age (exp(ln(120.72) + ln(1.053)×69 + ln(0.935)×69) / exp(ln(1.053)×69)).

**Table 4:** Mental health (MH) score regressions adjusted to sex, age, BMI, eGFR tercile, and comorbidities for constipation and constipation-related symptoms

Variable	Adjusted univariate analyses (row represents separate model)			Selected adjusted multiple regression (AIC 865.9, R^2^ = 0.352, *P* < 0.001)	
	Coefficient	*P* value	AIC	Coefficient (95% CI)	*P* value
**Frequency of defecation:**			886.2		
Once a day	reference	-		reference	-
Less than once a day	-15.35	<0.001		-10.63 (-18.96, -2.31)	0.013
More than once a day	-3.21	0.507		-0.08 (-9.25, 9.08)	0.986
**Discomfort in abdomen ^a^**	-17.31	<0.001	911.1	-14.07 (-21.69, -6.45)	<0.001
**Discomfort in abdomen:**			912.5	-	-
Lack	reference	-			
Mild	-15.41	<0.001			
Medium/severe	-19.50	<0.001			
**Pain in abdomen ^a^**	-16.18	<0.001	917.1	-	-
**Pain in abdomen:**			919.1	-	-
Lack	Reference	**-**			
Mild	-16.03	<0.001			
Medium/severe	-16.51	0.009			
**Bloating in abdomen ^a^**	-10.68	0.005	925.0	-	-
**Bloating in abdomen:**			924.1	-	-
Lack	reference	**-**			
Mild	-7.35	0.087			
Medium/severe	-15.90	0.002			
**Painful BM ^a^**	-13.97	0.013	926.8	-	-
**BM that was too small ^a^**	-11.36	0.013	926.8	-	-
**BM that was too small:**			926.9	-	-
Lack	reference	-			
Mild	-7.03	0.214			
Medium/severe	-16.86	0.008			
**Stomach cramps ^a^**	-10.98	0.014	926.9	-	-
**Straining/squeezing ^a^**	-9.94	0.011	926.39	-	-
**Straining/squeezing:**			926.35		
Lack	Reference	-			
Mild	-6.60	0.149			
Medium/severe	-14.22	0.005			
**Feeling false alarm ^a^**	-11.91	0.015	927.1		

Abbreviations: AIC: Akaike information criterion; BM: bowel movement. ^a^ presence of the symptom, regardless of its severity
