# Supplementary material for: Constipation and the Quality of Life in Conservatively Treated Chronic Kidney Disease Patients: A Cross-sectional Study
Source: Int J Med Sci. 2020 Oct 18;17(18):2954–63. doi: 10.7150/ijms.49648 (PMC7646105; doi:10.7150/ijms.49648)
Supplement: Supplementary file 1 — Supplementary tables. [file ijmsv17p2954s1.pdf]

Appendix

Tables S5-S10: Univariate analyses of constipation and constipation-related symptoms are adjusted to sex, age, BMI, eGFR tercile, and comorbidities. Selection of multiple regression model is described in the Methods section in the manuscript. [a] represents presence of the symptom, regardless of its severity.

Table S1. Internal consistency reliability and construct validity of SF-36v2 subscales

	Internal consistency reliability		Construct validity	
SF-36v2 subscale	Cronbach's alpha	Average inter-item correlation	Convergent validity [a]	Discriminant validity [b]
PF	0.934	0.587	0.65-0.80	0.32-0.68
RP	0.944	0.810	0.82-0.92	0.42-0.77
BP	0.916	0.846	0.87	0.41-0.66
GH	0.646	0.267	0.34-0.51	0.23-0.54
VT	0.834	0.557	0.61-0.71	0.40-0.65
SF	0.818	0.691	0.69	0.49-0.61
RE	0.962	0.893	0.92-0.93	0.42-0.72
MH	0.846	0.523	0.62-0.72	0.26-0.71

[a] Item-own scale correlation; poor convergent validity is indicated when items do not correlate .40 or higher with their hypothesized scale score. [b] Item-other scale correlation; poor discriminant validity is indicated when items correlate significantly higher with competing scales than with their hypothesized scale.

We found that nearly all subscales were reliable (Cronbach's alpha above 0.80), but General Health (GH) subscale was not. Average inter-item correlations exceeding 0.5 for the all rest of subscales proved reliability and suggested redundant character of some questions in the subscales. Moreover, convergent validity of the Polish version of SF-36v2 subscales, besides GH, was confirmed as all the correlation coefficients of items within these subscales exceeded the 0.40 criterion. Similarly, 4 out of 5 items in GH subscale and 1 item out of 5 items in MH subscale (MH05: happiness) failed their discriminant validity as their correlation coefficients with other scales were higher than correlation coefficients with their own subscales.

Abbreviations: BP, bodily pain; GH, general health perceptions; MH, general mental health; PF, physical functioning; RE, role limitations due to emotional problems; RP, role limitations due to physical health problems; SF, social functioning; VT, vitality.

Table S2. Pharmacological treatment of the study population.

	**All**	**Patients defecating less than once a day**	**Patients meeting criteria of functional constipation**	**Patients meeting Bristol scale criteria of constipation**
**N**<br>No data, n	111	38<br>5	21<br>0	28<br>14
Beta blockers, n (%)	75 (67.6)	25 (65.8)	14 (66.7)	19 (67.9)
Calcium channel blockers, n (%)	58 (52.3)	26 (68.4)	11 (52.4)	16 (57.1)
Diuretics, n (%)	60 (54.1)	23 (60.5)	9 (42.9)	21 (75.0)
NSAIDs, n (%)	24 (21.6)	7 (18.4)	2 (9.5)	4 (14.3)
Paracetamol, n (%)	14 (12.6)	8 (21.1)	5 (23.8)	4 (14.3)
Calcium supplements, n (%)	21 (18.9)	6 (15.8)	3 (14.3)	6 (21.4)
Vitamin D, n (%)	34 (30.6)	15 (39.5)	11 (52.4)	8 (28.6)
Iron, n (%)	8 (7.2)	2 (5.3)	1 (4.8)	3 (10.7)
Hypnotics, n (%)	7 (6.3)	1 (2.6)	2 (9.5)	1 (3.6)

Abbreviations: NSAIDs, nonsteroidal anti-inflammatory drugs.

Table S3. Poisson regression model showing variables significantly and independently associated with altered prevalence ratio of presence of at least 2 criteria of functional constipation.

Variable	Univariate analyses (row represents separate model)		Multiple regression	
	PR (95% CI)	*P* value	PR (95% CI)	*P* value
Use of vitamin D	2.30 (1.06, 5.00)	**0.035**	1.59 (0.76, 3.31)	0.214
Use of paracetamol	2.26 (0.97, 5.25)	0.058	2.67 (1.07, 6.64)	**0.035**
Use of NSAIDs	0.39 (0.10, 1.58)	0.188	0.34 (0.11, 1.00)	**0.049**
Age	1.02 (0.99, 1.04)	0.273	1.03 (0.99, 1.06)	0.152
Female sex	1.61 (0.72, 3.57)	0.239	2.10 (0.85, 5.23)	0.109
BMI	0.97 (0.91, 1.04)	0.369	1.02 (0.95, 1.08)	0.617
eGFR tercile:				
- High	reference	-	reference	-
- Medium	1.40 (0.41, 4.78)	0.590	0.79 (0.24, 2.61)	0.699
- Low	2.61 (0.91, 7.47)	0.074	2.85 (1.12, 7.28)	**0.028**
Diabetes	0.55 (0.20, 1.52)	0.252	0.37 (0.11, 1.23)	0.105
Heart failure	0.70 (0.22, 2.17)	0.534	0.67 (0.23, 2.00)	0.477
Hypothyroidism	1.35 (0.51, 3.55)	0.540	1.33 (0.55, 3.21)	0.522
Depression	2.31 (0.73, 7.32)	0.154	1.95 (0.74, 5.14)	0.177

Abbreviations: BMI, body mass index; eGFR, estimated glomerular filtration rate; NSAIDs, nonsteroidal anti-inflammatory drugs.

Table S4. Poisson regression model showing variables significantly and independently associated with altered prevalence ratio of having less than 7 bowel movements per week.

Variable	Univariate analyses (row represents separate model)		Multiple regression	
	PR (95% CI)	*P* value	PR (95% CI)	*P* value
Use of vitamin D	1.50 (0.89, 2.53)	0.129	1.38 (0.72, 2.65)	0.337
Use of paracetamol	1.84 (1.06, 3.18)	**0.030**	1.53 (0.72, 3.27)	0.271
Use of calcium channel blocker	2.00 (1.12, 3.56)	**0.018**	1.72 (0.98, 3.00)	0.059
Age	0.99 (0.98, 1.01)	0.417	0.99 (0.97, 1.01)	0.288
Female sex	1.70 (1.00, 2.90)	**0.049**	1.86 (0.96, 3.61)	0.066
BMI	0.98 (0.93, 1.04)	0.527	1.01 (0.95, 1.07)	0.845
eGFR tercile:				
- High	Reference	-	Reference	-
- Medium	1.50 (0.73, 3.09)	0.271	1.42 (0.70, 2.87)	0.334
- Low	1.67 (0.84, 3.31)	0.144	1.79 (0.87, 3.67)	0.114
Diabetes	0.87 (0.48, 1.57)	0.636	0.79 (0.40, 1.54)	0.484
Heart failure	0.75 (0.36, 1.56)	0.436	0.80 (0.37, 1.70)	0.559
Hypothyroidism	1.02 (0.51, 2.07)	0.949	0.80 (0.36, 1.76)	0.578
Depression	1.16 (0.38, 3.53)	0.787	0.68 (0.21, 2.20)	0.521

Abbreviations: BMI, body mass index; eGFR, estimated glomerular filtration rate.

Table S5. Adjusted physical functioning (PF) score regressions and selected multiple regression.

Variable	Adjusted univariate analyses (row represents separate model)			Selected multiple regression (AIC 903.0, $R^2 = 0.530$, $P < 0.001$)	
	Coefficient	*P* value	AIC	Coefficient (95% CI)	*P* value
Frequency of defecation: :			926.5		
- Once a day	reference	-		reference	-
- Less than once a day	-19.30	**<0.001**		-8.72 (-19.56, 2.12)	0.113
- More than once a day	-0.887	0.880		3.83 (-7.12, 14.79)	0.489
Discomfort in abdomen [a]	-13.70	**0.006**	970.6	-	-
Discomfort in abdomen:			972.3	-	-
- Lack	reference	-			
- Mild	-11.97	**0.048**			
- Medium/severe	-15.88	**0.017**			
Pain in abdomen [a]	-24.48	**0.001**	953.0	-19.28 (-29.51, -9.04)	**<0.001**
Pain in abdomen:			954.1	-	-
- Lack	reference	-			
- Mild	-26.76	**<0.001**			
- Medium/severe	-19.64	**0.009**			
BM that was too small [a]	-18.89	**0.001**	966.4	-11.08 (-21.88, -0.28)	**0.044**
BM that was too small:			964.7	-	-
- Lack	reference	-			
- Mild	-11.00	0.116			
- Medium/severe	-28.08	**<0.001**			
Stomach cramps [a]	-16.58	**0.002**	968.6	-	-
Stomach cramps:			970.1	-	-
- Lack	reference	-			
- Mild	-18.75	**0.003**			
- Medium/severe	-11.50	0.216			

Table S6. Adjusted role limitations due to physical health problems (RP) score regressions and selected multiple regression.

Variable	Adjusted univariate analyses (row represents separate model)			Selected multiple regression (AIC = 945.0, R^2 = 0.398, P < 0.001)	
	Coefficient	*P* value	AIC	Coefficient (95% CI)	*P* value
Frequency of defecation: :			963.0		
- Once a day	reference	-		Reference	-
- Less than once a day	-20.52	**<0.001**		-11.64 (-23.82, 0.54)	0.061
- More than once a day	-4.84	0.470		-1.71 (-14.53, 11.11)	0.792
Functional constipation	-19.35	**0.006**	1014.5	-	-
Discomfort in abdomen [a]	-15.63	**0.005**	1004.7	-	-
Discomfort in abdomen:			1005.5	-	-
- Lack	reference	-			
- Mild	-11.59	0.087			
- Medium/severe	-20.29	**0.005**			
Pain in abdomen [a]	-22.91	**<0.001**	996.4	-16.72 (-28.80, -4.64)	**0.007**
Pain in abdomen:			998.4	-	-
- Lack	reference	-			
- Mild	-22.46	**<0.001**			
- Medium/severe	-23.87	**0.007**			
BM that was too hard [a]	-14.41	**0.009**	1005.8	-	-
BM that was too hard			1005.0	-	-
- Lack	reference	-			
- Mild	-8.46	0.197			
- Medium/severe	-21.73	**0.003**			
BM that was too small:			1006.76	-	-
- Lack	reference	-			
- Mild	-5.17	0.510			
- Medium/severe	-24.58	**0.005**			

Stomach cramps [a]	-19.23	**0.002**	1002.6	-	-
Stomach cramps:<br>- Lack<br>- Mild<br>- Medium/severe	 reference<br>-21.17<br>-14.71	 -<br>**0.003**<br>0.159	1004.3	-	-
Straining/squeezing [a]	-16.76	**0.002**	1002.6	-9.358 (-19.86, 1.14)	0.080
Straining/squeezing:<br>- Lack<br>- Mild<br>- Medium/severe	 Reference<br>-17.54<br>-15.76	 -<br>**0.006**<br>**0.024**	1004.6	-	-

Table S7. Adjusted bodily pain (BP) score regressions and selected multiple regression.

Variable	Adjusted univariate analyses (row represents separate model)			Selected multiple regression model (AIC 987.6, $R^2 = 0.366$, $P < 0.001$)	
	Coefficient	*P* value	AIC	Coefficient (95% CI)	*P* value
Functional constipation	-16.17	**0.020**	1010.6	-	-
Bloating in abdomen [a]	-13.01	**0.012**	1000.2	-	-
Bloating in abdomen:			998.3	-	-
- Lack	reference	-			
- Mild	-7.71	0.185			
- Medium/severe	-21.31	**0.002**			
Pain in abdomen [a]	-19.75	**<0.001**	994.0	-16.03 (-27.11, -4.95)	**0.005**
Pain in abdomen:			995.8	-	-
- Lack	reference	-			
- Mild	-18.50	**0.005**			
- Medium/severe	-22.42	**0.010**			
BM that was too hard [a]	-14.07	**0.008**	999.4	-13.11 (-23.08, -3.14)	**0.010**
BM that was too hard			999.2	-	-
- Lack	reference	-			
- Mild	-8.95	0.160			
- Medium/severe	-20.37	**0.004**			
Discomfort in abdomen [a]	-14.31	**0.008**	999.4	-	-
Discomfort in abdomen:			998.6	-	-
- Lack	reference	-			
- Mild	-8.26	0.205			
- Medium/severe	-21.28	**0.002**			
Feeling false alarm:			1000.3		
- Lack	Reference	-		Reference	-
- Mild	-6.47	0.408		1.56 (-13.50, 16.62)	0.838
- Medium/severe	-29.45	**0.006**		-20.85 (-41.07, -0.64)	**0.043**

Table S8. Adjusted vitality (VT) score regressions and selected multiple regression.

Variable	Adjusted univariate analyses (each row represents separate model)			Selected adjusted regression (AIC 909.1, $R^2 = 0.373$, $P < 0.001$)	
	Coefficient	*P* value	AIC	Coefficient (95% CI)	*P* value
Discomfort in abdomen [a]	-13.62	**<0.001**	920.6	-10.78 (-18.24, -3.32)	**0.005**
Discomfort in abdomen:			920.1	-	-
- Lack	reference	-			
- Mild	-9.63	**0.042**			
- Medium/severe	-18.21	**<0.001**			
BM that was too hard			921.8		
- Lack	reference	-		reference	-
- Mild	4.69	0.314		7.34 (-1.36, 16.04)	0.097
- Medium/severe	-15.61	**0.002**		-12.66 (-22.06, -3.27)	**0.009**
Painful BM [a]	-12.98	**0.022**	928.0	-10.26 (-20.59, 0.06)	0.051
Bloating in abdomen [a]	-9.33	**0.015**	927.3	-	-
Bloating in abdomen:			927.0	-	-
- Lack	reference	-			
- Mild	-6.29	0.148			
- Medium/severe	-14.11	**0.006**			
Pain in abdomen [a]	-15.15	**<0.001**	919.5	-	-
Pain in abdomen:			921.5	-	-
- Lack	reference	-			
- Mild	-14.66	**0.002**			
- Medium/severe	-16.19	**0.012**			
Functional constipation	-10.25	**0.048**	938.6	-	-
Stomach cramps [a]	-11.98	**0.007**	925.7	-	-
Stomach cramps:			927.7	-	-
- Lack	reference	-			
- Mild	-11.56	**0.026**			
- Medium/severe	-12.95	0.090			

Feeling false alarm [a]	-12.49	**0.010**	926.6	-	-
Feeling false alarm:			926.8	-	-
- Lack	reference	-			
- Mild	-8.46	0.145			
- Medium/severe	-20.18	**0.011**			

Table S9. Adjusted social functioning (SF) score regressions and selected multiple regression

Variable	Adjusted univariate analyses (row represents separate model)			Selected adjusted multiple regression (AIC 982.0, $R^2 = 0.337$, $P < 0.001$)	
	Coefficient	*P* value	AIC	Coefficient (95% CI)	*P* value
Discomfort in abdomen [a]	-16.61	**0.001**	986.6	-	-
Discomfort in abdomen:			987.0		
- Lack	reference	-		Reference	-
- Mild	-12.36	**0.046**		-5.16 (-18.31, 8.00)	0.438
- Medium/severe	-21.50	**0.001**		-21.32 (-37.84, -4.80)	**0.012**
Pain in abdomen [a]	-18.84	**0.001**	985.1	-	-
Pain in abdomen:			985.3		
- Lack	reference	-		Reference	-
- Mild	-22.51	**0.000**		-16.44 (-29.95, -2.92)	**0.018**
- Medium/severe	-11.04	0.178		6.08 (-14.57, 26.73)	0.560
Painful BM [a]	-17.61	**0.016**	991.8	-	-
Straining/squeezing [a]	-12.21	**0.016**	991.7	-	-

Table S10. Adjusted role limitations due to emotional problems (RE) score regressions and selected multiple regression.

Variable	Adjusted univariate analyses (row represents separate model)			Selected adjusted regression (AIC 983.5, $R^2 = 0.359$, $P < 0.001$)	
	Coefficient	*P* value	AIC	Coefficient (95% CI)	*P* value
Discomfort in abdomen [a]	-16.99	**0.001**	987.0	-12.29 (-22.67, -1.90)	**0.021**
Discomfort in abdomen:			988.5	-	-
- Lack	reference	-			
- Mild	-14.60	**0.020**			
- Medium/severe	-19.74	**0.003**			
Painful BM [a]	-19.49	**0.008**	991.2	-13.22 (-27.37, 0.94)	0.067
BM that was too small [a]	-15.41	**0.009**	991.5	-9.11 (-20.75, 2.52)	0.123
Straining/squeezing [a]	-14.12	**0.005**	990.3	-	-
Straining/squeezing:			992.3	-	-
- Lack	Reference	-			
- Mild	-14.47	**0.016**			
- Medium/severe	-13.67	**0.037**			
